# Flexibility of Ballet Dancers in COVID-19 Pandemic: A Prospective Observational Study in Portugal

**DOI:** 10.3390/ijerph19159235

**Published:** 2022-07-28

**Authors:** Amanda Batista, Elmiro Neto, Luís Branquinho, Ricardo Ferraz, Joana Ribeiro, Pedro Forte, Lurdes Ávila-Carvalho

**Affiliations:** 1 Higher Institute of Educational Sciences of the Douro (ISCE Douro), 4560-708 Penafiel, Portugal; amandabatistagrd@yahoo.com.br (A.B.); luis.branquinho@iscedouro.pt (L.B.); joana.ribeiro@iscedouro.pt (J.R.); 2 CI-ISCE/ISCE Douro, 4560-708 Penafiel, Portugal; 3 Department of Physical Education, Federal University of Bahia, Salvador 40170-110, Bahia, Brazil; mironeto.personal@gmail.com; 4 Research Centre in Sports Sciences, Health Sciences and Human Development (CIDESD), 5001-801 Covilhã, Portugal; 5 Department of Sport Sciences, Instituto Politécnico de Bragança (IPB), 5300-253 Bragança, Portugal; ricardompferraz@gmail.com; 6 Department of Sports Sciences, University of Beira Interior (UBI), 6201-001 Covilhã, Portugal; 7 CIFI2D, Faculty of Sports (FADEUP), University of Porto, 4099-002 Porto, Portugal; lurdesavila2@gmail.com

**Keywords:** rhythmic gymnastics, COVID-19 pandemic, flexibility, functional asymmetry

## Abstract

The aim of this study was to verify how dancers’ flexibility work has developed during confinement through four assessment moments: before, during (two times), and after the lockdown period. The sample was formed by 18 dancers from the Porto Dance Conservatory (Portugal) with an average age of 11.4 ± 1.4 years and 1.4 ± 0.7 years of experience. To assess the passive and active flexibility level, we used seven of the International Gymnastics Federation’s recommended tests using main joints (i.e., hips and spine). The first evaluation was performed before the pandemic situation began in a training environment, and the second and third evaluation were performed during the lockdown, in home environment, and in virtual trainings. Finally, the last evaluation was carried out in a training environment after returning to face-to-face activities and with several rules such a social distancing and mask use. The results showed that significant improvements were verified in the flexibility level of the dancers from the first to the fourth moment of evaluation. In the current study, no statistical significance was noted for the decreased values of functional asymmetry between the preferred and non-preferred lower limbs. These differences may have substantial relevance for dancers’ harmonious body development.

## 1. Introduction

A severe acute respiratory syndrome outbreak caused by the Coronavirus 2 (SARSCoV-2 or COVID-19) occurred in December of 2019 in Wuhan, Hubei Province, China. The virus spread rapidly across the country and then to the whole world [1,2], causing a pandemic without precedent, leading to many restrictions on multiple areas of society, including sports and dance [3]. Many governments were forced to impose an almost global lockdown, with quarantine measures implemented for an indefinite time. The population was requested to stay at home, except for urgent or necessary outings [4,5].

In a short time, the world entered an extreme and unknown situation, in which people’s health became the main priority [4]. Therefore, the world of dance, had been affected by the effects of pandemic and confinement in an unprecedented way. Gradually, all dance contests and other events were postponed and almost all organized training or practice was banned [6].

In normal conditions, dancers have a high work volume [7], since classical ballet is a branch of art that combines a specific dance technique with a high level of physical requirement [8].

Dancers around the world had found themselves in a unique situation in which they were not only obliged to stop their usual athletic activity but were also confined to their homes, often without the usual space and motivation to keep to minimum training levels. Regardless of duration, isolation could have a significant impact on the physical state, well-being, and self-esteem of athletes [4,9].

Several consequences of isolation situation can have a short and long term negative effect on the athletes’ physical fitness and competitive performance [4]. There are direct organizational consequences for the dance practice: absence of organized training and competition, lack of appropriate communication between dancers and teachers, inability to move freely, lack of adequate exposure to sunlight, and improper training conditions [4,10]. Staying in quarantine still can have negative effects in lifestyle, through of, for example, a poor and inappropriate nutrition, poor sleep quality, insomnia, anxiety, addictions, depression, and loneliness [4,11]. Furthermore, this situation induces a probable physical inactivity that, consequently, cause to adverse physiological effects as increase in body fat and a decrease in muscle mass [4], opposite conditions to what is expected in dancers. Success in classical ballet is strongly influenced by visual appeal and body aesthetics, e.g., that dancers should have an appropriate body size and maintain low body fat [12].

Flexibility is considered one of main motor skills for successful professional realization of dancers [13], as it is necessary to have an extensive range of movement at their joints in order to effortlessly execute demanding choreography [14]. This motor capacity is developed through of regular and specialized training that includes a system of drills, stretching, and dance exercises [13].

The complexity of this pandemic scenario has limited the effectiveness of training methods. During the lockdown, training had to adapt to this unique situation in order to maintain the minimum physical and technical condition of dancers [3]. Problems that teachers faced include handling dancer needs, performance control, injuries, and other consideration related to training [9] in these new conditions, as well as difficulties maintaining motivation and work quality during training at home.

According to several authors [15,16], it is likely that during confinement, athletes had lost to 10% physical fitness for each week of total inactivity, given that physiological adaptation is a reversible process and that most of the aspects that determine it are lost during an extended period of inactivity.

Therefore, the aim of this study was to verify how dancers’ flexibility work has developed during confinement through four assessment moments: before, during (two times) and after the lockdown period. The study hypothesis is that the flexibility work performed at home through online classes causes significant improvements in dancers’ flexibility indices.

## 2. Materials and Methods

### 2.1. Participants

Eighteen dancers from the Porto Dance Conservatory (Portugal) aged between 10 and 14 years old (11.4 ± 1.4 years) and with 1.4 ± 0.7 years of experience (considering the date of 1st evaluation moment) volunteered to be part of the study. To calculate the sample size, the software G*Power 3.1 (Heinrich-Heine-Universität Düsseldorf, Düsseldorf, Germany) was used [17]. An a priori analysis was performed that determined that 15 subjects would be needed for the study (Effect size dz: 0.6, α error probability: 0.05, power: 0.85), However only 14 dancers volunteer for the study.

The study was approved by the scientific committee of the Higher Institute of Educational Sciences of the Douro, and all of the assessments were performed in accordance with the ethical standards of the Helsinki Declaration.

### 2.2. Study Design

To achieve the objectives of the study, a longitudinal and prospective observational study was used. A convenience sample of 18 dancers was analysed. The following inclusion criteria were used: (1) being a ballet dancer; (2) have carried out a flexibility assessment before the onset of the COVID-19 pandemic. As an exclusion criterion: (1) being a dancer in a type of dance other than ballet. During physical preparation classes, the dancers performed typical exercises essentially using elastic bands and chairs. Before the pandemic situation caused by COVID-19, physical preparation classes had a duration varying between 1–3 h 2 times a week. Due to confinement, there was an increase in the number of weekly stimuli (i.e., five times a week) for the same total duration of classes (i.e., variable from 1–3 h).

Thus, in order to verify the impact of increasing the number of weekly stimuli on dancers, four evaluations were carried out to access passive and active flexibility: one evaluation in the period before the pandemic in October 2019 (1st mom), three evaluations during the pandemic period in May 2020, February 2021 and June 2021 (second moment, third moment and fourth moment, respectively). The evaluations were carried out using a battery of tests validated in the literature to measure flexibility [18,19]. During the study, passive and active flexibility were considered dependent variables. The group subjected to the physical preparation classes was considered an independent variable.

### 2.3. Flexibility Assessment

**Figure 1:**
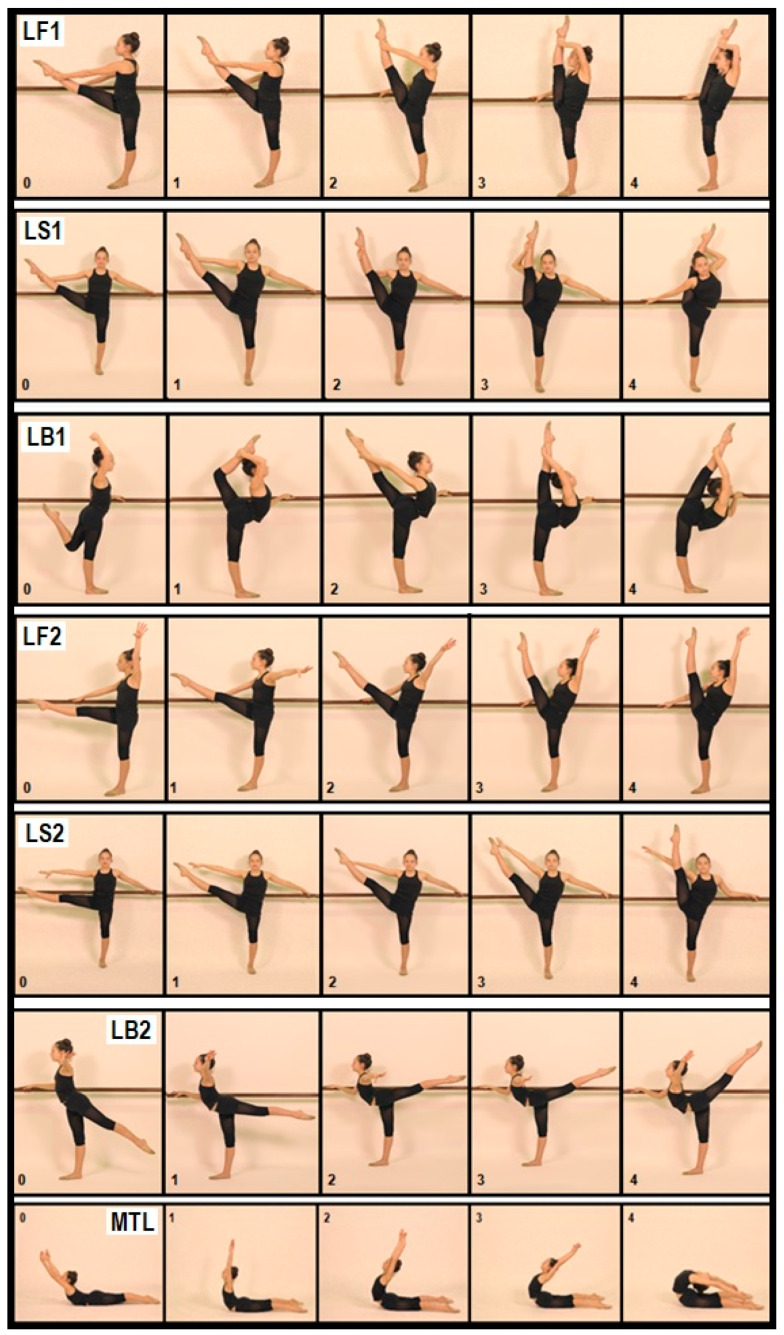
Reference points (0–4) of flexibility tests—Leg pull forward with help of the hand (LF1); sideways (LS1) or backward (LB1); leg raise forward (LF2), sideways (LS2) or backward—arabesque (LB2); maximum trunk lift (MTL).

To assess flexibility levels we used the International Gymnastics Federation recommended tests (Figure 1). This battery of tests presents simple methods of application and evaluation which were possible to perform in a home environment. Therefore, these tests were conducted in different moments and environments.

The first evaluation moment was carried out in October 2019 in ballet school, a training environment, before the pandemic situation began. A Nikon Photographic Camera and a Samsung Video Camera were used to register the images and videos. During the lockdown, in May 2020 (first quarantine in Portugal) and February 2021 (second quarantine in Portugal), we performed the second and third assessment moments, respectively, in the home environment in in a virtual training environment. The Zoom application was used to register the images and videos. Finally, the fourth evaluation moment was carried in June 2021, again at school, in training environment, after returning to face-to-face activities and with several rules such a social distancing and mask use. In this last evaluation moment, the same electronic equipment as in the first evaluation moment was used to capture images and videos.

All tests were executed following strictly the protocol proposed and analysed by two international gymnastics judges with more than 10 years of experience. At all evaluation moments, after one week, the judges repeated the analysis of images and videos. We observed high values of intra-examiner reliability (Kendall Coefficient of Concordance), ranged from 0.825 to 0.956, and inter-examiner reliability (Intraclass Correlation Coefficient), ranged 0.839 to 0.998, which confirms a high quality of information.

### 2.4. Flexibility Measurements

Maximum passive and active flexibility was measured in specific movements using the main joints (i.e., hip and spine). In total, seven tests were performed: leg pull forward with help of the hand (LF1), sideways (LS1) or backward (LB1); leg raise forward (LF2), sideways (LS2) or backward—arabesque (LB2); maximum trunk lift (MTL), to assess the flexibility comparing joint(s) range of motion against with an assessment chart (Figure 1). There are five levels attributed to each movement, referring to the maximum possible range of motion using a scale from 0 to 4 points (0 = poor, 1 = satisfactory, 2 = good, 3 = very good and 4 = excellent). Only integer numbers were attributed to results.

In the lower limb (LL) flexibility tests (L1 and L2), the dancers performed the exercises with preferred (PLL) and non-preferred lower limb (NPLL). The PLL is the leg that dancer prefers to perform the task and the NPLL is the support leg. A limit of 15% bilateral difference was established as the maximum value for a normal difference (i.e., no asymmetry) between PLL and NPLL (Marchetti, 2009). The asymmetry index (AI) was calculated using the equation [20]: AI (%) = [(PLL-NPLL)/PLL] × 100, where AI represents the asymmetry index, PLL is the preferred lower limb test result (mean value achieved in the active and passive flexibility tests with PLL) and NPLL the non-preferred lower limb test result (mean value achieved in the active and passive flexibility tests with NPLL).

The evaluation of functional asymmetry, by calculating the asymmetry index, is extremely relevant since it allows one to monitor the body’s harmonious development. The evident asymmetries may reflect negatively on the physical and technical preparation of gymnasts that, in the short term, become conditioning factors of performance and can produce pathologies in the long term [21].

### 2.5. Statistical Procedures

Statistical analysis was performed using the Statistical Package for Social Sciences (SPSS 27.0, IBM, Armonk, NY, USA). The significance level was set at 5%. Descriptive statistics were performed using the mean, standard deviation, median, minimum and maximum values. Were carried out non-parametric tests, due to the small sample size. Friedman test was applied to compare flexibility scores across four moments. Further, Wilcoxon test was used to compare the preferred and non-preferred body sides in all flexibility tests. For the present study, the confounding factor and interaction effects were assessed.

## 3. Results

Table 1 shows the four assessment moments in the passive and active flexibility tests. In the tests with lower limbs, the results were presented according to preferred (PLL) and non-preferred lower limbs (NPLL). Significant differences were observed in all variables. In the tests performed with PLL, we found differences in the first versus the fourth moment of evaluation in all passive and active flexibility tests (LF1, LS1, LB1, LF2, LS2 and LB2 tests). However, in the LB1 test also verified differences in the first versus second and third moments. In the flexibility tests performed with NPLL, the significant differences were observed in LF1, LF2, LS2 tests in the first versus third and fourth moments; LS1 and LB1 tests in the first versus second, third, and fourth moments; and LB2 test in the first versus fourth moment. Finally, the MTL test presented significant differences in the first versus third and fourth moments. We can highlight that all results in the fourth moment of evaluation in passive and active flexibility were significantly superior to the results verified in the first moment.

The LB1 tests with PLL and NPLL presented the lower-level results in all moments of evaluation since a high number of dancers could not perform the requested exercise, reaching the lowest level (0). However, we observed a constant improvement, although reduced, in the execution of this test during the evaluation moments.

Table 2 presents the analyse of asymmetry levels (PLL versus NPLL) in the passive and active flexibility tests in 1st and 4th moment of evaluation (before and after the confinement period).

Significant differences were found in PLL versus NPLL in most of the flexibility tests in the first and fourth moments of evaluation (LF1, LS1, LF2 and LS2). In the exercises with the lower limb lifted up backward (LB1 and LB2 tests), the dancers showed similar results in both lower limbs. According to the AI equation proposed by Chavett, Lafortune and Gray [20], the Table 2 presents the AI (%) for each of the flexibility tests in the first and fourth evaluation moments. No high levels of asymmetry were observed in the passive and active flexibility tests of leg raise backward with or without help of the hand (LB1 and LB2 tests). In contrast, we found functional asymmetry of different magnitudes in the passive and active flexibility tests of leg raise forward and sideways with or without help of the hand (LF1, LS1, LF2 and LS2 tests). However, although the AI decreased in all tests from the first to thr fourth moment (Table 2), these differences were not statistically significant: LF1 (*p* = 0.357); LS1 (*p* = 0.238); LB1 (*p* = 0.581); LF2 (*p* = 0.075); LS2 (*p* = 0.177) and LB2 (*p* = 0.905).

## 4. Discussion

The movement is the essence of classical dance technique, and must be learned and worked in the dance studio, where teachers’ examples and assistance are available. In addition, observation is very important in such as high demanding activity in order for it to be possible to work all movement perspectives. Understanding and successful execution of every detail of technical movement is essential to mastering the art of ballet [22]. Therefore, dance classes have particular requirements: the use of a broad space elapses from the progression of movements and choreographies, a floor with adequate characteristics for the use of pointe shoes, bar for performing specific exercises, and mirrors for immediate visual feedback and self-correction [23]. In contrast, the conditions found in home environment, are not in accordance with the needs of classical dance classes. In this way, during the confinement, the classical and contemporary dance classes were kept in an online format, but with a workload decrease coinciding with the recommendations made by several authors to conserve the specific technical skills training of modality [4,24]. As a complement, the physical preparation classes presented a considerable increase, from 2–3 h to 5 h per week. In these classes, the dancers worked especially the main motor capacities of classical ballet: flexibility, strength and endurance [13]. In this sense, it is possible to see positive aspects in the performed work during the lockdown period such as, for example, improvements in motor skills (as occurred in the flexibility levels of the dancers in our sample). We observed that the dancers presented higher passive and active flexibility levels after the lockdown period.

The fact that our sample is composed of dancers who practiced ballet (and flexibility training), on average, for only 1.4 years, provides a greater margin of improvement in this motor capacity compared to dancers who worked on flexibility for a longer time. However, the increase in the mentioned training volume seems to be the differentiating factor in this type of physical work.

Flexibility is one of main motor skills for successful professional performances of dancers [13] and, therefore, this work is essential to increase, even if not linearly, the possibility of executing different movements. The insufficient flexibility level hinders the body movements performance and makes it more difficult to perform it with perfect technique, to educate the expressiveness, and to demonstrate the movements’ lightness (which are all essential characteristics in dance) [21]. Several authors [7,8,12,13,14,25] emphasize the importance of developing this motor capacity in dance. However, according to Wyon et al. [14], although the passive flexibility is an important characteristic for all dancers, recent research has shown that active range of movement is a better predictor of dance performance especially in classical ballet and contemporary dance [26,27]. According to Grossman and Wilmerding [28], a high *développé* in dance, the combined actions of hip flexion, external rotation, and abduction, is considered a prerequisite in the world of professional dancer.

The improvements presented by the dancers in our study can be explained by the higher training volume, with physical preparation classes performed every day. According to Wyon [7], flexibility must be worked daily to generate significant adaptations. Furthermore, we can indicate other conditions that favoured daily work. In a normal classroom environment, the dancers of our study had little ability to concentrate, which caused high difficulties in achieving the desired evolution in the quality of movement. These difficulties are common, as the dancers of our sample are children and some with very little experience. On the other hand, in online classes, dancers were, in general, more concentrated, as they were unable to speak or look at their colleagues. Thus, in this lockdown period, besides increasing their workload, physical preparation classes became more regular and efficient. In the general analysis, through the comparison between the first and last moment of evaluation, the dancers in our study presented flexibility levels improvements in all specific movements assessed using main joints: spine and hip (exercises with PLL and NPLL). However, some tests indicated good and significant changes from the second moment (LB1 with PLL; LS1 and LB1 with NPLL), and others from the third moment of evaluation (LF1, LF2 and LS2 with NPLL; and MTL). These results show that the daily work had a positive and probably motivating effect since they could feel constant improvements in their flexibility levels. In addition, the asymmetry index was evaluated to determine the possible differences in flexibility levels with PLL and NPLL, and furthermore, to verify the effects of careful and organized bilateral work. Despite the natural asymmetry of the human body, it is suggested that the functional asymmetry in sports and dance is mainly the training result [21]. According to Lisitskaya [21], the apparent asymmetries may reflect negatively on the dancer’s physical and technical preparation, which in the short term become performance conditioning factors and in the long-term can produce pathologies. Therefore, the multilateral and harmonious development is essential in the flexibility work. Batista et al. [29] studied the flexibility asymmetry level in gymnasts of three different performance levels. The authors observed that the higher the performance level, the lower the asymmetry level in passive and active flexibility. Batista-Santos et al. [30] studied the flexibility level of the first division Portuguese gymnasts (13.7 ± 0.2 years). The first division in Portugal is formed by gymnasts of superior level. The authors verified high functional asymmetry levels in these gymnasts since 86.7% of the gymnasts presented differences equal or higher than 15% in flexibility level between LL. The authors highlighted two gymnasts with differences higher than 70%. In our study, initially, in the first moment of evaluation, according to recommended limit of 15% bilateral difference between PLL and NPLL [31], asymmetry levels were found to be normal in the LB1 and LB2 tests (11.1 and 12.5%, respectively) and different asymmetries magnitudes were found in the remaining tests—LF1, LS1, LF2 and LS2 (24.1–47.2%). Thus, after the period of lockdown and completion of the intended work (fourth moment), we observed a decreased of difference in passive and active flexibility level between PLL and NPLL in all tests performed, although without statistical significance (LF1: 33.3 to 25.9%; LS1: 24.1 to 16.7%; LB1: 11.1 to 4.2%; LF2: 42.2 to 25.5%; LS2: 36.1 to 18.5% and LB2: 12.5 to 11.6%). However, despite the fact that these differences were not statistically significant, we can consider these results and differences substantially relevant in the dancers’ harmonious body development. The LB1 test has an interesting characteristic since it is a movement that, in the first moment of evaluation, 88.9% (with PLL) and 94.4% (with NPLL) of the dancers were unable to perform the intended motor action (level 0 as result). Thus, the low level of asymmetry achieved in this test in the 1st moment of evaluation does not reflect the possible differences between PLL and NPLL but rather the inability to execute the intended movement.

In the last moment of evaluation, we observed that a smaller number of dancers achieved the lowest level in this test: 55.6% (with PLL) and 61.1% (with NPLL). The movement evaluated in the LB1 test requires high flexibility level using the spine, shoulders and hip joints, and therefore, its execution presents a high degree of complexity. Batista et al. [32] evaluated the flexibility level of elite Brazilian and Portuguese gymnasts with the same test used in our study. The authors verified that Portuguese gymnasts presented, in average, 4.0 and 3.0 points with PLL and NPLL, respectively, while the Brazilian gymnasts showed lower scores, 3.4 (PLL) and 1.3 (NPLL) points, and higher asymmetries levels. However, both groups showed higher results than the dancers in our sample. This movement is widely used in gymnastics (training and competition) and it is probably for this reason that gymnasts evidenced higher highlights in the results.

The LB2 test showed results with a low asymmetry level both in the first (12.5%) and in the last moment (11.6%) of evaluation. The motor action assessed in this test is a characteristic movement of classical ballet (leg raise backward in *arabesque*) and, in general, this movement is performed bilaterally in the dance choreographies. In the MTL test, we observed improvements in all evaluation moments, although the improvements with statistical significance were verified only from the first to the third and four moments. In this sense, the best result was found in the last moment of evaluation (2.4 ± 0.9 points). However, the dancers presented lower results compared to Brazilian (2.8 ± 1.1 points) and Portuguese (3.8 ± 0.5 points) elite gymnasts studied by Batista et al. [32]. In other study, Batista et al. [29] analysed the MTL test in Portuguese gymnasts of three different performance levels (Base, 1st division and Elite). The Elite level is composed only by junior and senior gymnasts, who are selected by the Gymnastics Federation of Portugal and form Portugal’s National Team. The Base level is constituted by lower-level gymnasts, while the 1st division gymnasts show a superior performance level. The results showed distinct scores: Base 1.7 ± 1.3 points; 1st division 3.2 ± 1.0 points and Elite 3.6 ± 0.7 points. Thus, the dancers in our sample only overcome the marks of the gymnasts of the lowest performance level. These results seem to reflect the higher importance e consequent higher workload of flexibility in gymnastics compared to ballet, especially at higher levels as Elite and 1st division.

Bobo-Arce et al. [3] studied how the training in rhythmic gymnastics had developed during the lockdown period. The authors verified that physical fitness was the content most used by gymnasts. In opposite, the technical sport contents, such as apparatus difficulties (many of them performed with high throws of the apparatus) and parts of the competitive routines (complex connections of body and apparatus movements performed on a floor area of 13 × 13 m) were less used. Several sports authors point out that during lockdown, they worked in particular on the physical fitness and sports skills according to sports requirements [4,33,34]. These completely new, different, and unexpected training conditions during the pandemic, added to the teachers’ inexperience a new reality imposed and many new challenges [35]. Although teachers generally have not had any prior experience with virtual training, many without adequate skills or technology, these professionals looked for home training alternatives to avoid possible effects of physical, technical, and psychological detraining. However, the situation was difficult and uncertain. For some dancers, the lockdown became an opportunity to experiment and work with power of imagination and new ways of exploration [36]. On the other hand, other dancers found themselves very unmotivated. Bobo-Arce et al. [3] observed that gymnasts of different performance levels left the sport due to the difficult circumstances of lockdown. For the elite gymnasts, the extrinsic factors, such as poor connectivity or lack of facilities and materials were the main reasons behind problems in training. On the other hand, for the lower-level gymnasts, intrinsic factors including the lack of contact with other gymnasts and the loss of sport goals were determinant.

In our study, no abandonment of dance practice was observed. However, the absence of shows, contests, working in a limited space, and the uncertainty of the confinement time were demotivating factors for more experienced dancers. While for the most novice dancers, the main reasons for discouragement seemed to be related to the lack of interaction with colleagues, as well as a lack of awareness about the importance of continuing work, even at home, and the uncertainties of face-to-face return.

The practice of dance and the physical training performed during the pandemic brought many challenges, a lot of learning, and new ways of seeing the movement and its possibilities. The effort and perseverance of teachers and dancers to overcome this pandemic and forced quarantine are already a great achievement.

## 5. Conclusions

Physical fitness, through physical preparation classes, was the item most used by dancers during the lockdown period. Thus, regular and intensive work brought significant improvements in passive and active flexibility levels. Furthermore (although without statistical significance) we observed a decreased of functional asymmetry levels between the preferred and non-preferred lower limb in all tests performed. These differences may have a significant impact in the dancers’ harmonious body development.

## 6. Study Limitations

Before confinement, the dancers in the sample had a different workload. However, with the implementation of online classes, the workload of physical preparation classes was the same for all groups of dancers. For the analysis, we did not consider the differences in the number of hours per week of work by dancers prior to confinement. Thus, the first moment of evaluation was analyzed as a starting point. However, this is a limitation of the study, since the previous working conditions may compromise the homogeneity of the sample.

The study showed differences in the degree of motivation during the confinement of dancer. Although these data are not assumptions but feedback from the dancers, this information was collected informally during the classes, without the application of a specific questionnaire. Thus, the data could have been formally collected and the results presented more precisely in order to complement the study performed.

The reduced sample size is a study limitation, since that the results should not be generalized and taken with some caution. The sample size is an important factor, but we consider it relevant to carry out the study even with a small sample to show the importance of flexibility work for dancers during the period of confinement. We could choose not to carry out the study given the small sample size, but we believe that this is a limiting factor but not a totally determining factor in the face of the objective. Finally, since this study is a prospective observational study without controlling for any covariates, it is necessary to interpret the results carefully.

## Figures and Tables

**Table 1:** Results and comparison of flexibility tests in the four moments of evaluation.

Flexibility Tests (in Points)			$1\mathrm{st}\;\mathrm{Mom}\;(\bar{x}\;\pm \;\mathrm{sd})$	$2\mathrm{nd}\;\mathrm{Mom}\;(\bar{x}\;\pm \;\mathrm{sd})$	$3\mathrm{rd}\;\mathrm{Mom}\;(\bar{x}\;\pm \;\mathrm{sd})$	$4\mathrm{th}\;\mathrm{Mom}\;(\bar{x}\;\pm \;\mathrm{sd})$	Proof Value	$\mathrm{All}\;\mathrm{Mom}\;(\bar{x}\;\pm \;\mathrm{sd})$
Passive Flexibility	PLL	LF1	2.1 ± 0.8	2.3 ± 0.8	2.6 ± 0.9	2.9 ± 0.8	*p* = 0.032 *^,c^	2.5 ± 0.8
		LS1	2.1 ± 0.9	2.4 ± 1.1	2.7 ± 1.1	2.7 ± 0.9	*p* ˂ 0.001 *^,c^	2.5 ± 1.0
		LB1	0.2 ± 0.4	0.7 ± 1.2	0.7 ± 1.0	1.0 ± 1.2	*p* ˂ 0.001 *^,a,b,c^	0.7 ± 0.9
	NPLL	LF1	1.4 ± 0.8	1.8 ± 0.8	2.1 ± 0.6	2.1 ± 0.7	*p* ˂ 0.001 *^,b,c^	1.8 ± 0.6
		LS1	1.6 ± 0.8	2.3 ± 0.7	2.2 ± 0.6	2.2 ± 0.7	*p* ˂ 0.001 *^,a,b,c^	2.1 ± 0.4
		LB1	0.1 ± 0.3	0.4 ± 0.7	0.7 ± 1.0	0.9 ± 1.2	*p* ˂ 0.001 *^,a,b,c^	0.5 ± 0.7
Active Flexibility	PLL	LF2	1.3 ± 1.0	1.7 ± 0.8	1.8 ± 0.9	2.2 ± 0.8	*p* ˂ 0.001 *^,c^	1.7 ± 0.8
		LS2	1.6 ± 0.9	2.0 ± 1.1	2.3 ± 1.0	2.3 ± 0.9	*p* = 0.002 *^,c^	2.0 ± 0.8
		LB2	1.9 ± 1.1	2.2 ± 1.0	2.1 ± 0.9	2.4 ± 0.8	*p* = 0.040 *^,c^	2.1 ± 0.8
	NPLL	LF2	0.6 ± 1.0	1.0 ± 0.8	1.6 ± 0.8	1.7 ± 0.9	*p* ˂ 0.001 *^,b,c^	1.2 ± 0.8
		LS2	0.9 ± 0.8	1.4 ± 0.9	1.7 ± 1.0	1.8 ± 1.0	*p* ˂ 0.001 *^,b,c^	1.4 ± 0.6
		LB2	1.6 ± 0.9	1.7 ± 1.0	1.9 ± 1.0	2.1 ± 0.6	*p* = 0.021 *^,c^	1.8 ± 0.7
	MTL		1.4 ± 0.6	1.9 ± 0.9	2.2 ± 0.9	2.4 ± 0.9	*p* ˂ 0.001 *^,b,c^	2.0 ± 0.8

Note: $\bar{x}$: mean; sd: standard deviation; mom: moment; PLL: preferred lower limb; NPLL: non-preferred lower limb; * *p* ≤ 0.05: significant differences—^a^ 1st moment versus 2nd moment; ^b^ 1st moment versus 3rd moment; ^c^ 1st moment versus 4th moment. Flexibility tests (in points): Leg pull forward with help of the hand (LF1); sideways (LS1) and backward (LB1); Leg raise forward (LF2), sideways (LS2) and backward—arabesque (LB2); Maximum trunk lift (MTL).

**Table 2:** Asymmetry levels in flexibility tests in first and fourth moments of evaluation.

Flexibility Tests (in Points)		1st Moment				4th Moment			
		$\mathrm{PLL}\;(\bar{x}\;\pm \;\mathrm{sd})$	$\mathrm{NPLL}\;(\bar{x}\;\pm \;\mathrm{sd})$	Proof Value	AI (%)	$\mathrm{PLL}\;(\bar{x}\;\pm \;\mathrm{sd})$	$\mathrm{NPLL}\;(\bar{x}\;\pm \;\mathrm{sd})$	Proof Value	AI (%)
Passive Flexibility	LF1	2.1 ± 0.8	1.4 ± 0.8	*p* = 0.008 *	33.3%	2.9 ± 0.8	2.1 ± 0.7	*p* ˂ 0.001 *	25.9%
	LS1	2.1 ± 0.9	1.6 ± 0.8	*p* = 0.008 *	24.1%	2.7 ± 0.9	2.2 ± 0.7	*p* = 0.003 *	16.7%
	LB1	0.2 ± 0.4	0.1 ± 0.3	*p* = 0.945	11.1%	1.0 ± 1.2	0.9 ± 1.2	*p* = 0.194	4.2%
Active Flexibility	LF2	1.3 ± 1.0	0.6 ± 1.0	*p* = 0.006 *	47.2%	2.2 ± 0.8	1.7 ± 0.9	*p* = 0.013 *	25.5%
	LS2	1.6 ± 0.9	0.9 ± 0.8	*p* = 0.010 *	36.1%	2.3 ± 0.9	1.8 ± 1.0	*p* = 0.046 *	18.5%
	LB2	1.9 ± 1.1	1.6 ± 0.9	*p* = 0.257	12.5%	2.4 ± 0.8	2.1 ± 0.6	*p* = 0.414	11.6%

Note: $\bar{x}$: mean; sd: standard deviation; PLL: preferred lower limb; NPLL: non-preferred lower limb; AI: Asymmetry index; * *p* ≤ 0.05: significant differences; Lower limb flexibility tests (in points): Leg pull forward with help of the hand (LF1); sideways (LS1) and backward (LB1); Leg raise forward (LF2), sideways (LS2) and backward—arabesque (LB2).
